# Examinations for the Uncertificated

**Published:** 1888-01-21

**Authors:** 


### THE NURSING MIRROR.

Communications for this column should be addressed The Mirror, care of the Editor, 38, Old Jewry, London, E.C.

### EXAMINATIONS FOR THE UNCERTIFICATED.

"M. B." writes:

"May I be allowed to trouble you with a suggestion that I think would prove of great service to the nursing world in general, and more especially to the large number of nurses who do not hold certificates. Many small hospitals do not grant them, and in those that do it too often only means that the nurse has worked a certain time (sometimes only a few months) in the hospital, and that during that time she has had an opportunity of seeing medical and surgical work, and that her conduct has been satisfactory. The certificates granted by some of the large London hospitals mean much more than this, but as they do not take unlimited probationers, a large number of nurses are left practically uncertificated.

"Would it not be possible to establish in connexion with the registration of nurses an examination that would entitle them to a certificate from The Hospitals Association, in the same way as certificates are granted by the Obstetrical Society to midwives?

"I would suggest that there should be three examinations:

"1. On nursing only, including all that is required for private nursing.

"2. On ward management, for those who wished to become charge nurses or ward sisters.

"3. On hospital domestic management, for those who wished to work as matrons or lady superiors.

"No nurse to be eligible for the second or third examinations who has not passed the first.

"Surely a certificate granted by The Hospitals Association would acquire in time a higher value than one from any hospital.

"If my suggestion is useless, accept my apologies for troubling you."

